# Premorbid BMI as a prognostic factor in small-cell lung cancer—a single institute experience

**DOI:** 10.18632/oncotarget.24935

**Published:** 2018-05-15

**Authors:** Cho-Hao Lee, Chin Lin, Chieh-Yung Wang, Tzu-Chuan Huang, Yi-Ying Wu, Wu-Chien Chien, Jia-Hong Chen

**Affiliations:** ^1^ Division of Hematology and Oncology Medicine, Department of Internal Medicine, Tri-Service General Hospital, National Defense Medical Center, Taipei, Taiwan, Republic of China; ^2^ School of Public Health, National Defense Medical Center, Taipei, Taiwan, Republic of China; ^3^ Department of Research and Development, National Defense Medical Center, Taipei, Taiwan, Republic of China; ^4^ Division of Pulmonary and Critical Care Medicine, Department of Internal Medicine, Tri-Service General Hospital, National Defense Medical Center, Taipei, Taiwan, Republic of China; ^5^ Graduate Institute of Clinical Medicine, College of Medicine, Taipei Medical University, Taipei, Taiwan, Republic of China

**Keywords:** overweight, small-cell lung cancer, prognostic factor, body mass index

## Abstract

Numerous evidence has indicated that excess weight is associated with an increased risk of mortality in patients in several cancer types including breast, colorectal, pancreatic, endometrial, and prostate cancer However, with respect to non-small cell lung cancer and upper aero-digestive cancer, evidence suggests that low body mass index (BMI) may increase the risk of mortality of these cancers, but a definitive link between premorbid BMI and overall survival in small cell lung cancer patients has yet to be fully explored. To investigate this possibility, we conducted a retro-spective of 173 small-cell lung cancer patients. Multivariate Cox analysis indicated that pretreatment overweight (BM I ≥ 23) was an independent prognostic factor for overall survival (OS) (Hazard ratio, = 0.58, 95% CI = 0.39–0.87, *p* = 0.008). In addition, meta-regression revealed that per-formance status (≤ 2) marginally interacted with increased BMI (*p* = 0.068). However, subgroup analysis showed that patients with a BMI ≥ 23 and performance status ≤ 2 had the best OS (Hazard ratio: 0.31, 95% CI: 0.16–0.61, *p* = 0.001). Premorbid BMI and performance status level are easy to measure and may provide physicians an additional measurement to predict a small-cell lung cancer patient’s survival. The data from the present study indicates that a, further large scale prospective study is warranted to better assess the association of pretreatment BMI and OS in small-cell lung cancer.

## INTRODUCTION

Small-cell lung cancer (SCLC) accounts for 15% of all lung cancers and is a highly aggressive tumor that is marked by early metastases and poor prognosis. Approximately 60–75% of patients with SCLC are diagnosed at later stages of disease progression [1–3]. While SCLC patients appear initially responsive to chemotherapy, most patients show cancer progression within months. Thus, research is urgently needed to identify predictive factors associated with improved prognosis. Previously, a meta-analysis study conducted by Wang *et al.* (2017) [4] revealed the association between body mass index (BMI) and mortality in non-small cell lung cancer (NSCLC) patients.

More than 50% of patients with advanced cancer have been diagnosed as having malnutrition despite having to meet a high nutritional demand due to an altered metabolic rate [5, 6]. Nearly 20% of cancer patients die of malnutrition induced by their tumors or from side effects from their treatments [7], and malnourished patients are at higher risk of poor prognosis and outcomes [8, 9]. Thus, the utility of a patient’s nutritional status—such as the rate of anemia [10] and BMI before treatment [11]—as a prognostic marker has been previously studied [12–14]. In addition, inflammation has been shown to play a prominent role in tumor progression and metastasis [15]. Inflammation factors, such as C-reactive protein, neutrophil-to-lymphocyte ratio (NLR) and platelet-to-lymphocyte ratio have extensively been characterized poor prognostic factor in various types of tumors [16–20].

Epidemiological studies have observed a significant inverse relationship between BMI and lung cancer incidence [21, 22]. Yun (2016) (*N* = 809) found BMI > 23 compared with BMI < 23 had significant lower risk of all-caused mortality (HR: 0.57, 95%CI: 0.38–0.86) in lung cancer patients [23]. In addition, a recent meta-analysis showed that being underweight was a risk factor for lung cancer, while excess weight provided a potential protective effect [24]. However, this meta-analysis did not assess the relationship specifically between BMI and mortality in SCLC patients. While many observational studies examined the relationship between BMI and mortality in patients with NSCLC, to the best, of our knowledge, such associations have not been extensively studied for SCLC, only reviewed in Inomata *et al.* (2016) [25]. In the present study, we evaluated nutritional status, inflammation markers, eastern cooperative oncology group performance status (ECOG PS), and basic patient data for their potential use as a available prognostic marker SCLC. Thus, the aim of our study was to retrospectively investigate the association between BMI and overall survival (OS) of SCLC patients, using data collected solely at Tri-Service General Hospital/National Defense Medical Center (TSGH/NDMC).

## RESULTS

### Baseline characteristics

A total of 30 (17.3%) males and 143 (82.7%) females with SCLC were included in the present study. The clinical/pathological characteristics of the 173 patients are summarized in Table 1. There were 8 patients still alive at the end of follow-up. The median follow-up time was 34.62 months. Among these patients, 2 (1.1%) were diagnosed as stage 1; 2 (1.1%) were stage 2; 39 (22.5%) were stage 3; and 130 (75.1%) were stage 4 SCLC. The baseline demographic, clinical, and laboratory characteristics of the patients’, including their BMI: < 23 (*n* = 110) or ≥ 23 (*n* = 63), are summarized in Table 1. The mean age of our participants was 69.25 and 67.05 years for males and females, respectively. More than half of the patients (*n* = 103; 59.5%) had PS of 0–2. Totally 126 patients received intravenous chemotherapy at least 1 cycle; among them, 63 patients received cisplatin plus etoposide, 33 patients carboplatin plus etoposide and 30 patients received chemo-radiotherapy. 1 patient received local radiotherapy and 1 patient received surgery. BMI > 23BMI < 23Patient with PS 3 and PS 4 received best supportive care accounted for 51.5% and 87.5% respectively Significant differences were observed in clinical stage, Charlson comorbidity index (CCI), ECOG PS, and levels of platelet (PLT), albumin, and alkaline phosphatase (ALP) (*p* < 0.05). The OS and Progression-Free Survival (PFS) were also significantly longer in patients with BMI ≥ 23 (OS, *p* < 0.001; PFS, *p* = 0.001).

**Table 1:** Basic characteristic

Variable	BMI > 23 (*N* = 63)	BMI < 23 (*N* = 110)	*p*–value
Stage			0.005^*^
Stage : 1	2 (3.2%)	0 (0.0%)	
Stage : 2	0 (0.0%)	2 (1.8%)	
Stage : 3	21 (33.3%)	18 (16.4%)	
Stage : 4	40 (63.5%)	90 (81.8%)	
Age (year)	67.05 ± 12.17	69.25 ± 11.51	0.238
Gender			0.199
Female	49 (77.8%)	94 (85.5%)	
Male	14 (22.2%)	16 (14.5%)	
Body Mass Index (BMI)	27.18 ± 3.19	20.59 ± 2.45	<0.001^*^
Metastases			0.055
No Metastases	25 (39.7%)	26 (23.6%)	
Organ Metastases : 1	23 (36.5%)	43 (39.1%)	
Organs Metastases > 1	15 (23.8%)	41 (37.3%)	
Charlson Comorbidity Index (CCI)	7.71 ± 2.43	8.63 ± 2.22	0.013^*^
Chief Complain (CC)			0.072
Respiratory system	57 (90.5%)	88 (80.0%)	
Other	6 (9.5%)	22 (20.0%)	
ECOG PS			0.001^*^
PS : 0	9 (14.5%)	5 (4.7%)	
PS : 1	28 (45.2%)	31 (29.2%)	
PS : 2	13 (21.0%)	17 (16.0%)	
PS : 3	7 (11.3%)	26 (24.5%)	
PS : 4	5 (8.1%)	27 (25.5%)	
WBC (UL)	8705.71 ± 3713.11	9113.73 ± 4415.66	0.537
Neutrophil (%)	69.77 ± 10.80	72.17 ± 11.27	0.174
Absolute Neutrophil Count	6240.78 ± 3433.25	6849.91 ± 4202.10	0.329
Lymphocyte (%)	19.76 ± 9.64	18.51 ± 8.96	0.395
Absolute Lymphocyte Count	1607.14 ± 859.81	1490.71 ± 779.07	0.364
Neutrophil/Lymphocyte Ratio	7.18 ± 19.81	9.51 ± 9.34	0.456
Hemoglobin (Hgb, g/Dl)	12.57 ± 2.15	12.48 ± 2.10	0.784
Platelet (PLT, 10^3/uL)	228.30 ± 72.86	261.55 ± 113.84	0.039^*^
C-Reactive Protein (mg/L)	7.63 ± 9.67	12.50 ± 39.42	0.366
Lactic Dehydrogenase (LDH, IU/L)	399.53 ± 217.44	799.42 ± 2039.28	0.232
Albumin (g/Dl)	3.68 ± 0.55	3.46 ± 0.58	0.016^*^
Alkaine Phosphatase (ALP, U/L)	81.50 ± 34.88	128.59 ± 163.97	0.039
Numbers of patients received intravenous chemotherapy (at least 1 cycle)	49 (77.8%)	77 (70.0%)	0.077
Cycles of first-line chemotherapy	4.67 ± 2.22	4.38 ± 2.14	0.479
Pericardia Effusion^**$**^			0.499
Large	2 (25.0%)	2 (14.3%)	
Small	3 (37.5%)	3 (21.4%)	
Minimal	3 (37.5%)	9 (64.3%)	
Non-Smoking Patients	14 (22.2%)	31 (28.9%)	0.346
Smoking (Pack per year)	468.3 ± 231.9	446.6 ± 230.3	0.809
Progression Free Survival (day)	218 (192–264)	176.5 (133–216)	0.001^*^
The numbers of survivors at the end of data cut-off	6 (9.5%)	2 (1.8%)	0.005^*^
Overall Survival (day)	341 (281–468)	226 (157–301)	<0.001^*^

Abbreviation: ECOG PS: Eastern cooperative oncology group performance status; WBC: white blood count.

blood count.

^$^The pericardia effusion was measured by cardiac echography. We defined pericardial effusion as small if a circumferential echo-free space is smaller than 0,5 cm (<100 ml), moderate effusion if echo-free space is about 1 cm (100–500 ml), and large if echo free space is more than 1 cm (>500 ml).

^*^P-value < 0.05.

### Analysis of factors related to OS

**Figure 1:**
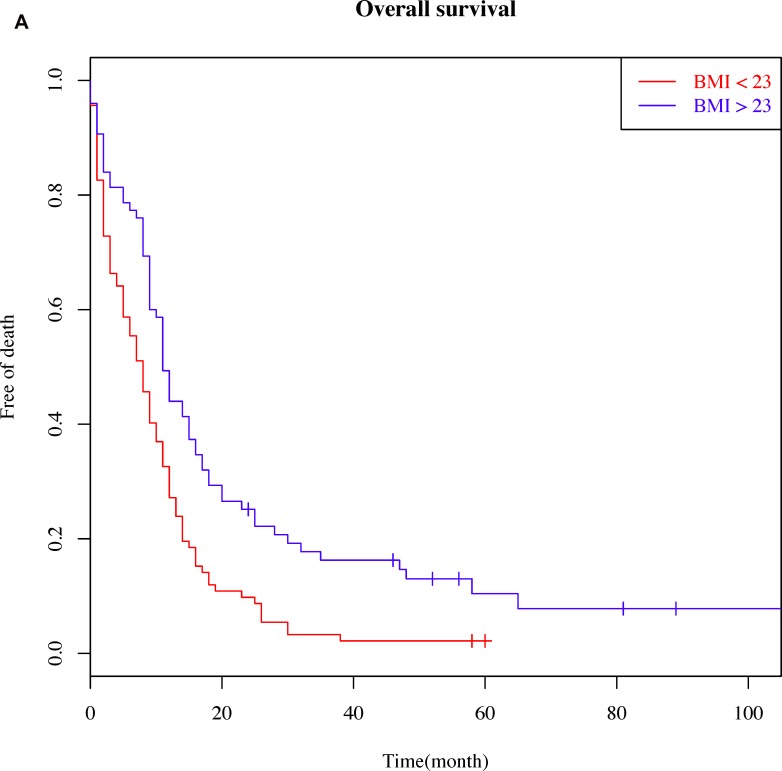

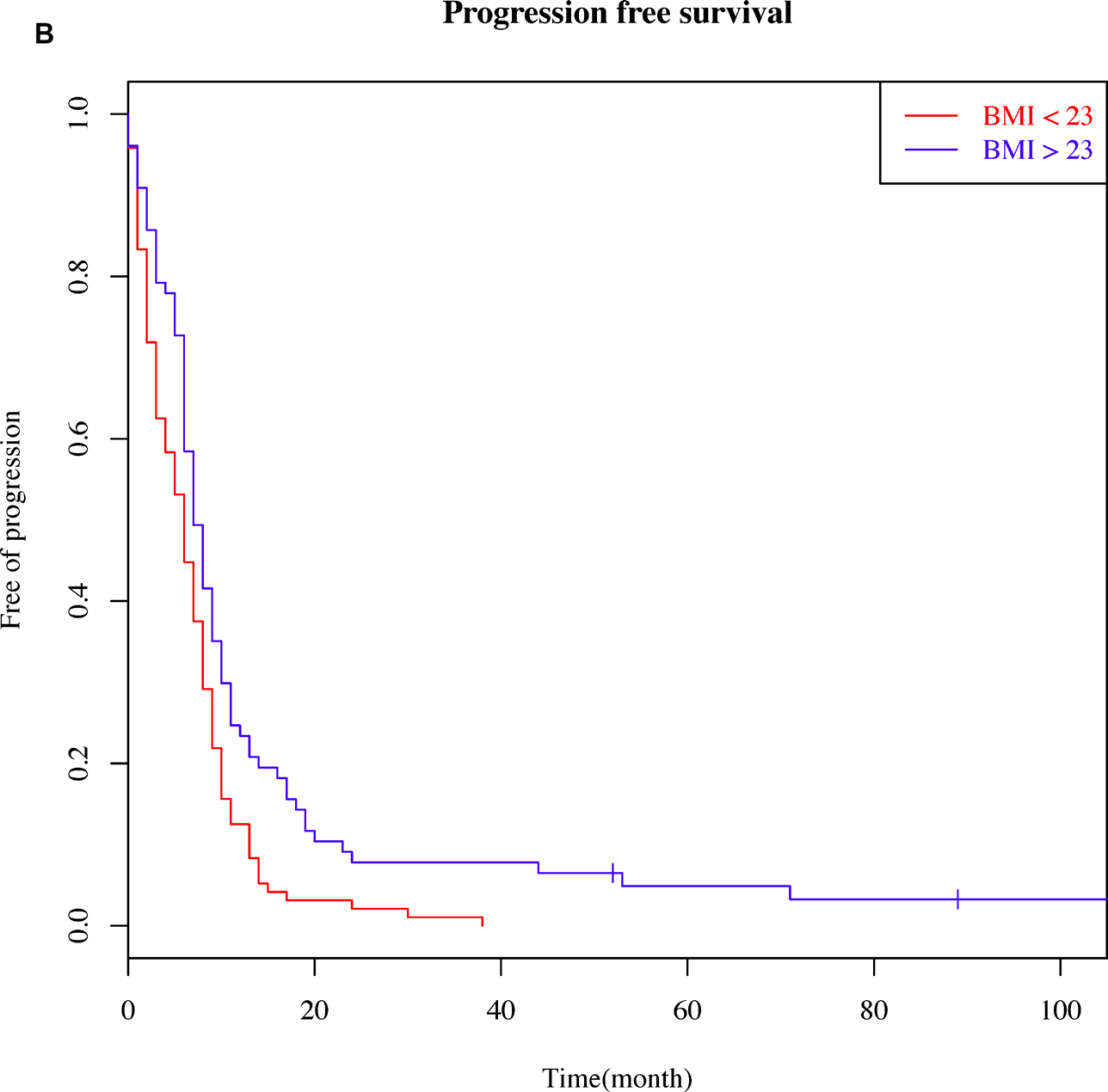
Patient Survival Curves (Kaplan–Meier survival plots) (**A**) with respect to body mass index (BMI) on overall survival (BMI cut-off value of 23) and (**B**) with respect to BMI on disease-free survival (BMI cut-off value of 23).

Correlations between OS and candidate prognosis factors were analyzed by univariate analyses (Table 2). We observed significant associations between OS and clinical stage, mean age, overweight, PS, white blood count (WBC), NLR, albumin, and ALP levels. The OS was higher in the BMI ≥ 23 group compared to the BMI < 23 group (620.0 vs. 311.7 days, *p* < 0.001, Figure 1A). Furthermore, patients who were overweight exhibited longer PFS (421.3 vs. 200.9 days, *p* = 0.001, Figure 1B).

**Table 2:** Univariate factor

Independent variable	HR (95% CI)	*p*-value
Stage		0.049^*^
Stage : 1–3	1.00	
Stage : 4	1.40 (1.00–1.95)	0.049
Age		0.001^*^
Age < 70 years	1.00	
Age ≥ 70 years	1.63 (1.22–2.19)	0.001^*^
Gender		0.208
Female	1.00	
Male	0.78 (0.53–1.15)	0.208
Body Mass Index		<0.001^*^
BMI < 23	1.00	
BMI ≥ 23	0.56 (0.40–0.77)	<0.001^*^
Metastases		0.992
No Metastases	1.00	
Organ metastasis : 1	1.02 (0.72–1.46)	0.902
Organs metastases > 1	1.01 (0.70–1.47)	0.951
Complication		0.445
No complication	1.00	
Complication	1.16 (0.79–1.71)	0.445
Charlson Comorbidity Index (CCI)	1.03 (0.97–1.10)	0.328
Chief Complain (CC)		0.135
Respiratory system	1.00	
Other	1.36 (0.91–2.02)	0.135
ECOG PS		<0.001^*^
PS : 0–2	1.00	
PS : > 2	2.06 (1.51–2.83)	<0.001^*^
WBC (UL)		0.007^*^
WBC < 4500	1.00	
WBC within normal	1.72 (0.84–3.51)	0.140
WBC ≥ 11000	2.71 (1.27–5.79)	0.010^*^
NLR		0.004^*^
NLR < 3.04	1.00	
NLR ≥ 3.04	1.54 (1.14–2.06)	
PLT (10^3/uL)	1.00 (1.00–1.00)	0.132
Albumin (g/dL)		0.024^*^
Albumin > 3.5	1.00	
Albumin ≤ 3.5	1.42 (1.05–1.93)	
ALP (U/L)		0.001^*^
AL*P* < 104	1.00	
ALP ≥ 104	1.90 (1.30–2.76)	
Chemotherapy cycles	1.01 (0.94–1.09)	0.743
Smoking (pack per year)		0.786
Smoking < 182.5	1.00	
Smoking ≥182.5	0.94 (0.58–1.52)	

Abbreviation: BMI: Body Mass Index; Charlson comorbidity index; CC: Chief Complain; ECOG PS: Eastern cooperative oncology group performance status; WBC: white blood count; NLR: Neutrophil/Lymphocyte Ratio; PLT: levels of platelet; ALP: alkaline phosphatase.

^*^P-value < 0.05.

### Cox proportional hazards models for multiple factors associated with OS

The prognostic effect of clinicopathologic variables associated with OS is summarized in Table 3. In model 1, we adjusted for the basic characteristic variables that were significantly different between the BMI ≥ 23 and BMI < 23 groups. In model 2, we adjusted for the variables that were significantly different as indicated in Tables 1 and Table 2 (i.e., disease stage, age, gender, BMI, metastases, CCI, WBC, PS, NLR, albumin, PLT, ALP levels, complication, and smoking). Model 3 is the final result of a further multivariate analysis, and demonstrated that, BMI ≥ 23 (Hazard Ratio (HR) = 0.45, 95% Confidence Interval (CI) = 0.31–0.79, *p* = 0.004); age ≥ 70 years (HR = 2.90, 95 % CI = 1.69–4.98, *p* < 0.001), CCI < 8 (HR = 0.45, 95% CI = 0.26–0.79; *p* = 0.005); WBC ≥ 11000/μL (HR = 3.70, 95% CI=1.05–13.03, *p* = 0.042); ECOG PS > 2 (HR = 1.93, 95% CI=1.15–3.24, *p* = 0.013); and smoking ≥182.5 pack per year (HR = 2.32, 95% CI = 1.13–4.76, *p* = 0.019) independently predicted OS (Table 3), after adjusting for stage, age, gender, BMI, CCI, WBC, PS, NLR, albumin, PLT, and ALP levels and we furthermore adjusted additional four variables (respiratory chief complain, metastasis to organs, complications, and smoking), that were deemed not statistically significant in the univariate analyses but were still clinically meaningful risk factors.

**Table 3:** Multivariate factors

	Model 1		Model 2		Model 3	
Independent variable	HR (95% CI)	*p*-value	HR (95% CI)	*p*-value	HR (95% CI)	*p*-value
Stage : 4	1.40 (1.00–1.95)	0.049	1.55 (0.91–2.66)	0.106	1.53 (0.82–2.83)	0.180
Age ≥ 70 years			1.66 (1.07–2.57)	0.024	2.90 (1.69–4.98)	<0.001^*^
Female					0.59 (0.33–1.07)	0.082
BMI ≥ 23	0.56 (0.40–0.77)	<0.001	0.69 (0.46–0.91)	0.041	0.45 (0.31–0.79)	0.004^*^
Metastases > 1 organs					1.20 (0.74–1.96)	0.456
CCI < 8	0.97 (0.72–1.30)	0.850	0.58 (0.36–0.93)	0.025	0.45 (0.26–0.79)	0.005^*^
WBC ≥ 11000(UL)			1.88 (0.64–5.52)	0.251	3.70 (1.05–13.03)	0.042^*^
CC of Respiratory system					1.74 (0.92–3.30)	0.090
ECOG PS > 2	2.06 (1.51–2.83)	<0.001	1.80 (1.14–2.83)	0.011	1.93 (1.15–3.24)	0.013^*^
NLR ≥ 3.04			1.00 (0.65–1.53)	0.995	0.88 (0.53–1.46)	0.625
Albumin ≤ 3.5 (g/dL)	1.42 (1.05–1.93)	0.024	0.77 (0.48–1.21)	0.254	0.75 (0.44–1.25)	0.269
PLT ≤ 150 or ≥ 450 (10^3/uL)	1.14 (0.76–1.70)	0.532	1.21 (0.69–2.12)	0.515	1.68 (0.77–3.71)	0.195
Complication^$^					1.68 (0.91–3.08)	0.096
ALP ≥ 104 (U/L)	1.90 (1.30–2.76)	0.001	1.34 (0.80–2.26)	0.263	1.19 (0.65–2.19)	0.577
Smoking ≥ 182.5 (Pack per year)					2.32 (1.13–4.76)	0.022^*^

Abbreviation: BMI: Body Mass Index; CCI: Charlson comorbidity index; WBC: white blood count; CC: chief complaint; ECOG PS: Eastern cooperative oncology group performance status; NLR: Neutrophil/Lymphocyte Ratio; PLT: levels of platelet; ALP: alkaline phosphatase.

^$^Superior vena cava syndrome, syndrome of inappropriate antidiuretic hormone secretion, Cushing’s syndrome.

^*^P-value < 0.05.

**Figure 2:**
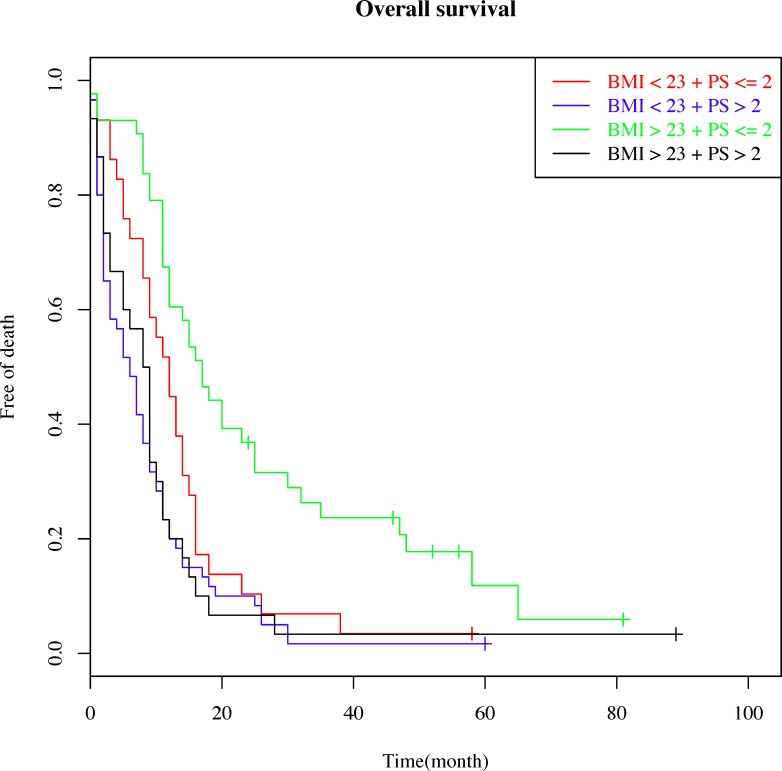
Patient survival curves showing the interaction between overall survival and two prognostic factors: the body mass index (BMI) and performance status (PS) Patients were divided into 4 groups according to the 2 prognostic factors: a) BMI < 23 and PS ≤ 2; b) BMI < 23 and PS > 2; c) BMI > 23 and PS ≤ 2; and d) BMI > 23 and PS > 2.

We evaluated the interaction between BMI and PS with regards to OS; for this analysis, all patients were divided into 4 groups according to the 2 prognostic factors: group A (BMI < 23 and PS ≤ 2), group B (BMI < 23 and PS > 2), group C (BMI ≥ 23 and PS ≤ 2), and group D (BMI ≥ 23 and PS > 2). Survival curves for each group are shown in Figure 2. The median OS of group A, group B, group C, and group D were 12.1 months, 6.2 months, 17.3 months, and 9.1 months, respectively. When PS ≤ 2 (group A and group B), the survival of patients with BMI ≥ 23 was significantly higher than that of patients with BMI < 23 (log-rank P <0.001). No survival difference was observed between patients with BMI < 23 or BMI ≥ 23 when PS > 2 (group C vs. group D, log-rank *P* = 0.062). BMI ≥ 23 and PS ≤ 2 (group C) had the best OS (HR = 0.31, 95% CI = 0.16–0.61, *P* < 0.05). The above results imply that there was a marginal quantitative interaction between PS and BMI, although this interaction was not significant in the final Cox-regression model (*p* = 0.068) (Table 4).

**Table 4:** Adjusted Interaction of BMI and PS

Stratified variable	ECOGPS	Adj-HR (95% CI)	*P*-value	*P*-value (interaction)
BMI				0.068
BMI < 23	PS > 2	1.00		
	PS ≤ 2	0.58 (0.29–1.15)	0.120	
BMI > 23	PS > 2	1.00		
	PS ≤ 2	0.31 (0.16–0.61)	0.001^*^	

Abbreviation: BMI: Body Mass Index; ECOG PS: Eastern cooperative oncology group performance status;

^*^P-value < 0.05.

## DISCUSSION

To the best of our knowledge, only Inmate *et al.* 2016 reported the lactate dehydrogenase (LDH) level and BMI maybe useful prognostic factors in SCLC patient [25]. Our present study provides more evidence to explore the association of overweight BMI with clinicopathological variables and survival outcome in SCLC patients of Asia population.

Lung cancer remains the top cause of cancer-related death worldwide [26]. Compared to NSCLC, SCLC is more aggressive and relapses early, and despite its higher initial sensitivity to chemotherapy, the prognosis remains poor for patients with this disease [27]. Over the past few decades, some potential surrogate markers for SCLC have been studied and reported; however, prognosis has been challenging to predict [28–31], underscoring the urgent need for useful factors that can differentiate the prognosis of various patient populations.

Our study found that SCLC patients who were overweight prior to treatment had better OS than their normal and underweight counterparts (*p* < 0.001). A deeper multivariate analysis also identified that being overweight was an independent prognostic factor for SCLC patients. Excess weight was associated with an increased risk of mortality in patients with several other cancer types, including breast, colorectal, pancreatic, endometrial, and prostate cancer [32–34]. However, with respect to lung and upper aero-digestive cancer, low BMI may actually increase the risk of mortality [24, 35].

Wang *et al.* (2017) [4] conducted a meta-analysis to explore the association between BMI and OS in all kinds of lung cancer. When patients were stratified by ethnicity, Asians with elevated BMI had higher OS than Westerners. Compared with normal-weight patients, underweight patients had reduced lung cancer-specific survival as well as lower OS. However, among the 51 cohorts studied, 50 were of NSCLC and only one of SCLC [25].

Consistent with studies of NSCLC patients [9], we found that OS and PFS were higher in SCLC patients whose BMI was ≥ 23 compared to those whose BMI was < 23 (OS: 620.0 vs. 311.7 days, *p* < 0.001; PFS: 421.3 vs. 200.9 days, *p* = 0.001, Figure 1A and 1B). In contrast, there was no statistically significant relationship between the BMI and age, gender, stage, CCI, WBC, NLR, albumin, PLT, and ALP. Multivariate Cox analysis indicated that being overweight prior to treatment was an independent prognostic factor for long-term outcome (HR = 0.58, 95% CI = 0.39–0.87, *p* = 0.008) (Table 3).

These data indicate that pending future validation, increased BMI has the potential to be an easily measurable prognostic indicator. A marginal interaction between PS and BMI was also noted (*p* = 0.068). Patients with PS ≤ 2 and overweight (BMI ≥ 23) had the longest OS (*p* < 0.05). This result needs to be validated due to the small sample size available for our study.

The mechanisms underlying the relationship between BMI and mortality in patients with lung cancer is still not clearly understood. Patients with respiratory diseases can exhibit weight loss over many years, contributing to their mortality; perhaps alluding to the negative impact of reduced weight on lung cancer patient survival [36]. In addition, weight gain may imply better PS among lung cancer patients [37]. Yet another possibility is that the inverse association between BMI and mortality risk may reflect differences between tobacco smokers and non-smokers. Nicotine activates the melanocortin axis in the brain, suppressing the appetite and reducing food intake [35].

The present study had the following limitations: the single institutional, retrospective design, small population size and population heterogeneity. We are also worried about that selection bias may make it difficult to generalize the results of our analysis and the relative small population failed to detect any significant difference between groups and influential factors.

These clinical trials should investigate the association of pretreatment body weight of SCLC patients with OS, while controlling for important potential confounding factors, such as sex, age, treatment, lifestyle, and disease duration. Also, as a retrospective study, we were not able to assess pretreatment overweight in a prospective cohort to fully assess its role as a prognostic factor in SCLC. Where possible, the effect of weight change over the course of treatment on SCLC patient survival of patients should be evaluated.

In conclusion, being overweight prior to diagnosis is associated with improved survival and in combination of well performance status are superior prognostic factors of patients with SCLC based on our single institute experience. However, the selection biases maybe existed due to retrospective design, further studies are eagerly needed. The pretreatment BMI and PS can be more easily measured than clinical cancer stage, serum biomarker, gene mutation, suggesting that BMI may provide an additional simple and convenient prognostic factor for OS in SCLC patients. Furthermore, this may help inform decision making in the clinic. Finally, the maintenance of adequate body weight is likely to increase a lung cancer patient’s life-span.

## MATERIALS AND METHODS

### Population selection

A total of 260 patients who had histologically-confirmed SCLC from January 2000 to March 2012, and treated at the Tri-Service General Hospital/National Defense Medical Center (TSGH/NDMC) were evaluated for eligibility. Patients with pretreatment laboratory values and physical measurement information were included, while patients with incomplete follow-up data or body weight data were excluded. Follow-up information was collected from hospitalization records or from family contact. A total of 173 eligible patients were enrolled for the evaluation.

### Data collection

The detailed clinical characteristics including BMI, smoking history, age, gender, PS, disease stage, therapeutic strategies, and survival were obtained for medical records. Serum laboratory data were retrospectively collected based on pretreatment evaluation from the medical records. The time to measure body weight is within 10 days before diagnosis. Besides, if there were any body weight changes in 10 days, we took the average body weight as premorbid data. The cut-off value for the BMI was set at 23, which was adjusted to the Asia-Pacific population according to the World Health Organization Expert consultation [38]. We used the tumor-node-metastasis cancer staging system by the American Joint Committee on Cancer to classify the cancer. The OS was defined as time from the date of diagnosis to the date of death. The PFS was defined as a period from the first day of diagnosis until documented objective tumor progression or death. The date of follow up cut-off was March 25, 2017.

### Statistical analysis

Continuous variables were described using mean ± SD and the categorical variables were analyzed by a Chi-squared test. The optimal cut-off values were determined using time-dependent receiver operating curve (ROC) analysis. Time-dependent ROC analysis was performed using R software, version 3.2.3 and the ‘time ROC’ package [39]. The Kaplan–Meier method and log-rank tests were employed to compare the survival curves. Multivariate analyses were conducted to identify significant independent prognostic factors for the prognosis. Hazard ratio (HR) for each factor was calculated using a Cox-regression proportional hazards model, and median OS was calculated using Kaplan–Meier analysis. Multivariate analysis was performed using factors satisfying *p*-values less than 0.05 from the univariate analysis. A *p*-value less than 0.05 was used as the cut-off value for statistical significance. Software “R” version 3.2.3 was used for the statistical analysis.
